# Poly[tetra­kis(μ_4_-4,6-dimethyl-5-nitro­benzene-1,3-dicarboxyl­ato-κ^2^ *O* ^1^:*O* ^1′^:*O* ^3^:*O* ^3′^)bis­(pyridine-κ*N*)dizinc]

**DOI:** 10.1107/S1600536811013092

**Published:** 2011-04-13

**Authors:** Qing-Yu Ma, Rui-Fang Guan, Guo-Zhong Li

**Affiliations:** a School of Materials Science and Engineering, University of Jinan, Jinan 250100, People’s Republic of China

## Abstract

In the title complex, [Zn_2_(C_10_H_7_NO_6_)_2_(C_5_H_5_N)_2_]_*n*_, the repeat unit is a centrosymmetic tetra-carboxyl­ato-*O,O*’-bridged dimer in which each Zn^II^ atom is five-coordinated by four O atoms from different dianionic 4,6-dimethyl-5-nitro­iso­phthalate ligands [Zn—O = 2.0283 (18)–2.0540 (19) Å] and one N atom from a pyridine mol­ecule [Zn—N = 2.030 (2) Å] in the axial site of a slightly distorted square-pyramidal coordination sphere. The Zn⋯Zn separation is 2.9750 (6) Å. The complex dimers are extended into a two-dimensional polymeric structure parallel to (100) through bridges provided by the second carboxyl­ate group of the ligand.

## Related literature

For the structure of a similar but discrete tetra-carboxyl­ato-bridged Zn_2_ dimer, see: Yu *et al.* (2011 ▶).
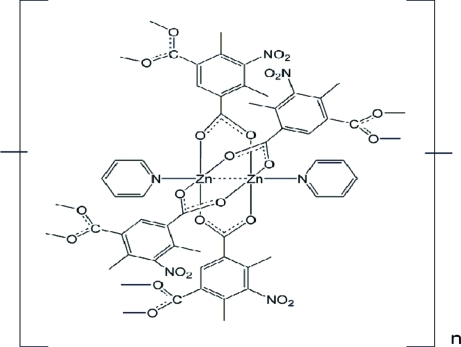


## Experimental

### 

#### Crystal data

[Zn(C_10_H_7_NO_6_)(C_5_H_5_N)]
*M* *_r_* = 381.64
Monoclinic, 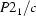

*a* = 10.2947 (9) Å
*b* = 11.8526 (10) Å
*c* = 12.8501 (11) Å
β = 97.933 (2)°
*V* = 1553.0 (2) Å^3^
*Z* = 4
Mo *K*α radiation
μ = 1.62 mm^−1^
*T* = 298 K
0.12 × 0.10 × 0.08 mm

#### Data collection

Bruker APEXII CCD area-detector diffractometer
Absorption correction: multi-scan (*SADABS*; Sheldrick, 1996 ▶) *T*_min_ = 0.824, *T*_max_ = 0.879
8899 measured reflections
3525 independent reflections
2750 reflections with *I* > 2σ(*I*)
*R*_int_ = 0.029

#### Refinement

*R*[*F* ^2^ > 2σ(*F* ^2^)] = 0.033
*wR*(*F* ^2^) = 0.081
*S* = 1.03
3525 reflections
217 parameters
H-atom parameters constrained
Δρ_max_ = 0.43 e Å^−3^
Δρ_min_ = −0.43 e Å^−3^

### 

Data collection: *APEX2* (Bruker, 2007 ▶); cell refinement: *SAINT-Plus* (Bruker, 2007 ▶); data reduction: *SAINT-Plus*; program(s) used to solve structure: *SHELXS97* (Sheldrick, 2008 ▶); program(s) used to refine structure: *SHELXL97* (Sheldrick, 2008 ▶); molecular graphics: *SHELXTL* (Sheldrick, 2008 ▶); software used to prepare material for publication: *SHELXTL*.

## Supplementary Material

Crystal structure: contains datablocks global, I. DOI: 10.1107/S1600536811013092/zs2089sup1.cif

Structure factors: contains datablocks I. DOI: 10.1107/S1600536811013092/zs2089Isup2.hkl

Additional supplementary materials:  crystallographic information; 3D view; checkCIF report

## Figures and Tables

**Fig. 1.**
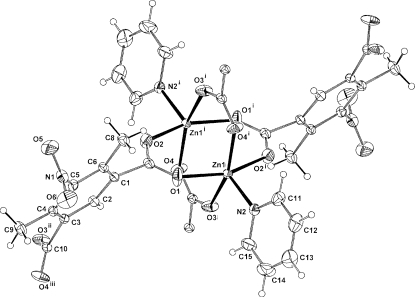
The atom numbering scheme for the centrosymmetric dimeric unit in the title compound with displacement ellipsoids drawn at the 30% probability level. Symmetry codes are: (i) -x+1, -y, -z + 1; (ii) -x + 1, y - 1/2, -z - 3/2; (iii) x, -y + 1/2, z + 1/2. The second set of hydrogen atoms on the 50% rotationally-disordered methyl groups (C8 and C9) are omitted.

**Fig. 2.**
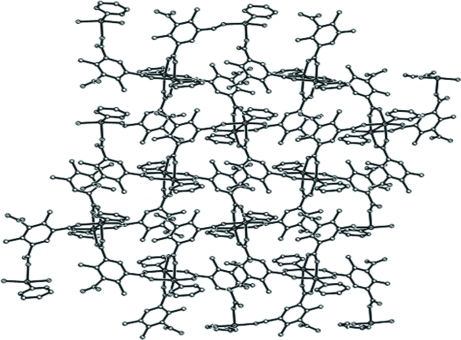
The two-dimensional polymeric extension of the title compound viewed down the a axial direction.

**Fig. 3.**
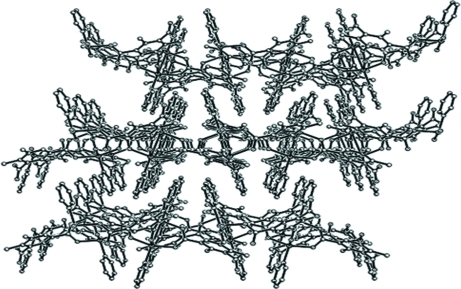
The packed structure of the title compound viewed down the c axial direction.

## supplementary crystallographic information

### Comment

The title complex [Zn_2_(C_10_H_7_NO_6_)_2_(C_5_H_5_N)_2_]_n_ (I) was obtained by chance when we were attempting to prepare some porous metal-organic framework materials by the reaction of 4,6-dimethyl-5-nitroisophthalic acid with Zn(NO_3_)_2_. In (I), the repeating unit is a centrosymmetic tetra-carboxylato *O,O*' bridged dimer (Fig. 1) in which each Zn^II^ is five-coordinated by four O atoms from different dianionic 4,6-dimethyl-5-nitroisophthalate ligands [Zn—O, 2.0283 (18)–2.0540 (19) Å] and one N atom from a pyridine molecule [Zn—N, 2.030 (2) Å] in the axial site of a slightly distorted square pyramidal coordination sphere. Each of the Zn atoms has an approximately square-pyramidal environment, with four O atoms in a plane and the pyridine N atom at the apical site. The Zn—Zn separation in the dimer is 2.9750 (6) Å. This dimeric unit is similar to that found in the structure of the tetra-benzoato-bridged but discrete Zn_2_ dimer with 4-(dimethylamino)pyridine (Yu *et al.*, 2011).  The complex dimers in (I) are extended into a two-dimensional polymer structure through bridges provided by the second carboxylate group of the ligand (Figs. 2, 3).

### Experimental

4,6-Dimethyl-5-nitroisophthalic acid (2 mg, 0.0046 mmol) and Zn(NO_3_)_2_ (4 mg, 0.0137 mmol) were dissolved in 1 ml of a mixed DMSO–H_2_O solvent (2:1) and 2 drops pyridine were added. This solution was sealed in a Pyrex glass tube and heated to 120° C over a period of 10 h, and then maintained at this temperature for 50 h, after which it was cooled to room temperature over 17 h. Colourless rod crystals were obtained, which were filtered, washed with water and dried in air.

### Refinement

All H atoms were placed in calculated positions (C—H = 0.93–0.96 Å) and refined in a riding mode with *U*_iso_(H) = 1.2*U*_eq_(C). The methyl H atoms are rotationally disordered over six equal half-occupancy sites.

### Crystal data

**Table:**

[Zn(C_10_H_7_NO_6_)(C_5_H_5_N)]	*F*(000) = 776
*M**_r_* = 381.64	*D*_x_ = 1.632 Mg m^−^^3^
Monoclinic, *P*2_1_/*c*	Mo *K*α radiation, λ = 0.71073 Å
Hall symbol: -P 2ybc	Cell parameters from 2968 reflections
*a* = 10.2947 (9) Å	θ = 2.3–26.1°
*b* = 11.8526 (10) Å	µ = 1.62 mm^−^^1^
*c* = 12.8501 (11) Å	*T* = 298 K
β = 97.933 (2)°	Rod, colourless
*V* = 1553.0 (2) Å^3^	0.12 × 0.10 × 0.08 mm
*Z* = 4	

### Data collection

**Table:**

Bruker APEXII CCD area-detector diffractometer	3525 independent reflections
Radiation source: fine-focus sealed tube	2750 reflections with *I* > 2σ(*I*)
graphite	*R*_int_ = 0.029
φ and ω scans	θ_max_ = 27.7°, θ_min_ = 2.0°
Absorption correction: multi-scan (*SADABS*; Sheldrick, 1996)	*h* = −8→13
*T*_min_ = 0.824, *T*_max_ = 0.879	*k* = −15→15
8899 measured reflections	*l* = −16→16

### Refinement

**Table:**

Refinement on *F*^2^	Primary atom site location: structure-invariant direct methods
Least-squares matrix: full	Secondary atom site location: difference Fourier map
*R*[*F*^2^ > 2σ(*F*^2^)] = 0.033	Hydrogen site location: inferred from neighbouring sites
*wR*(*F*^2^) = 0.081	H-atom parameters constrained
*S* = 1.03	*w* = 1/[σ^2^(*F*_o_^2^) + (0.0353*P*)^2^ + 0.6981*P*] where *P* = (*F*_o_^2^ + 2*F*_c_^2^)/3
3525 reflections	(Δ/σ)_max_ = 0.001
217 parameters	Δρ_max_ = 0.43 e Å^−^^3^
0 restraints	Δρ_min_ = −0.43 e Å^−^^3^

### Special details

**Table:**

Geometry. Bond distances, angles etc. have been calculated using the rounded fractional coordinates. All su's are estimated from the variances of the (full) variance-covariance matrix. The cell esds are taken into account in the estimation of distances, angles and torsion angles
Refinement. Refinement of *F*^2^ against ALL reflections. The weighted *R*-factor *wR* and goodness of fit *S* are based on *F*^2^, conventional *R*-factors *R* are based on *F*, with *F* set to zero for negative *F*^2^. The threshold expression of *F*^2^ > σ(*F*^2^) is used only for calculating *R*-factors(gt) *etc*. and is not relevant to the choice of reflections for refinement. *R*-factors based on *F*^2^ are statistically about twice as large as those based on *F*, and *R*- factors based on ALL data will be even larger.

### Fractional atomic coordinates and isotropic or equivalent isotropic displacement parameters (Å^2^)

**Table:**

	*x*	*y*	*z*	*U*_iso_*/*U*_eq_	Occ. (<1)
Zn1	0.63668 (3)	−0.03544 (2)	0.53898 (2)	0.0233 (1)	
O1	0.65410 (19)	0.13708 (16)	0.54695 (18)	0.0546 (8)	
O2	0.4496 (2)	0.18909 (15)	0.49411 (17)	0.0508 (7)	
O3	0.55390 (18)	−0.03094 (18)	0.67374 (14)	0.0454 (7)	
O4	0.6491 (2)	−0.02213 (19)	0.38329 (14)	0.0499 (7)	
O5	0.7497 (2)	0.62087 (19)	0.42547 (16)	0.0600 (8)	
O6	0.9095 (2)	0.5865 (2)	0.5453 (2)	0.0703 (10)	
N1	0.7959 (2)	0.57613 (18)	0.50579 (17)	0.0352 (7)	
N2	0.8232 (2)	−0.07998 (17)	0.59645 (16)	0.0298 (6)	
C1	0.5998 (2)	0.32743 (18)	0.56737 (17)	0.0245 (7)	
C2	0.6751 (2)	0.39863 (19)	0.51257 (17)	0.0253 (7)	
C3	0.7112 (2)	0.50127 (19)	0.56086 (18)	0.0258 (7)	
C4	0.6795 (2)	0.53868 (18)	0.65714 (17)	0.0253 (7)	
C5	0.5997 (2)	0.46553 (19)	0.70674 (17)	0.0235 (6)	
C6	0.5600 (2)	0.36307 (19)	0.66100 (17)	0.0260 (7)	
C7	0.5647 (3)	0.2080 (2)	0.53197 (18)	0.0283 (7)	
C8	0.7134 (3)	0.3664 (2)	0.4076 (2)	0.0382 (9)	
C9	0.7241 (3)	0.6519 (2)	0.7019 (2)	0.0416 (9)	
C10	0.5618 (2)	0.48883 (19)	0.81384 (17)	0.0259 (7)	
C11	0.9056 (3)	−0.1198 (3)	0.5336 (2)	0.0455 (10)	
C12	1.0338 (3)	−0.1460 (3)	0.5712 (3)	0.0625 (14)	
C13	1.0764 (3)	−0.1350 (3)	0.6760 (3)	0.0739 (15)	
C14	0.9916 (4)	−0.0970 (4)	0.7408 (3)	0.0817 (14)	
C15	0.8655 (3)	−0.0695 (3)	0.6982 (2)	0.0559 (11)	
H6A	0.50520	0.31680	0.69400	0.0310*	
H8A	0.67890	0.29300	0.38790	0.0570*	0.500
H8B	0.80720	0.36510	0.41230	0.0570*	0.500
H8C	0.67830	0.42060	0.35570	0.0570*	0.500
H8D	0.76410	0.42610	0.38270	0.0570*	0.500
H8E	0.63580	0.35410	0.35830	0.0570*	0.500
H8F	0.76460	0.29850	0.41490	0.0570*	0.500
H9A	0.69250	0.66260	0.76810	0.0620*	0.500
H9B	0.69000	0.71040	0.65410	0.0620*	0.500
H9C	0.81820	0.65490	0.71220	0.0620*	0.500
H9D	0.77470	0.68930	0.65480	0.0620*	0.500
H9E	0.77710	0.64150	0.76880	0.0620*	0.500
H9F	0.64890	0.69700	0.71080	0.0620*	0.500
H11A	0.87570	−0.13000	0.46260	0.0550*	
H12A	1.09060	−0.17090	0.52580	0.0750*	
H13A	1.16230	−0.15320	0.70300	0.0890*	
H14A	1.01850	−0.08980	0.81260	0.0980*	
H15A	0.80810	−0.04270	0.74230	0.0670*	

### Atomic displacement parameters (Å^2^)

**Table:**

	*U*^11^	*U*^22^	*U*^33^	*U*^12^	*U*^13^	*U*^23^
Zn1	0.0253 (2)	0.0240 (2)	0.0210 (1)	0.0011 (1)	0.0044 (1)	0.0003 (1)
O1	0.0406 (12)	0.0231 (10)	0.0991 (18)	0.0015 (9)	0.0057 (12)	−0.0082 (10)
O2	0.0471 (12)	0.0278 (10)	0.0708 (14)	−0.0077 (9)	−0.0156 (10)	−0.0060 (9)
O3	0.0346 (10)	0.0791 (15)	0.0245 (9)	0.0051 (10)	0.0108 (8)	0.0017 (9)
O4	0.0510 (12)	0.0788 (15)	0.0218 (9)	0.0216 (11)	0.0114 (9)	0.0125 (9)
O5	0.0789 (16)	0.0588 (14)	0.0439 (12)	−0.0111 (12)	0.0142 (11)	0.0230 (11)
O6	0.0460 (14)	0.0838 (18)	0.0819 (18)	−0.0243 (13)	0.0113 (13)	0.0200 (15)
N1	0.0456 (14)	0.0298 (11)	0.0338 (12)	−0.0076 (10)	0.0185 (10)	−0.0013 (9)
N2	0.0285 (11)	0.0286 (10)	0.0324 (11)	0.0014 (9)	0.0047 (9)	−0.0001 (9)
C1	0.0281 (12)	0.0225 (11)	0.0229 (11)	−0.0012 (9)	0.0036 (9)	−0.0028 (9)
C2	0.0302 (12)	0.0246 (12)	0.0220 (11)	0.0007 (10)	0.0064 (9)	−0.0024 (9)
C3	0.0298 (13)	0.0227 (11)	0.0261 (12)	−0.0020 (9)	0.0077 (10)	0.0036 (9)
C4	0.0317 (13)	0.0204 (11)	0.0239 (11)	−0.0007 (10)	0.0043 (9)	−0.0005 (9)
C5	0.0278 (12)	0.0243 (11)	0.0189 (10)	0.0017 (10)	0.0047 (9)	−0.0002 (9)
C6	0.0303 (13)	0.0243 (12)	0.0244 (12)	−0.0048 (9)	0.0079 (10)	0.0010 (9)
C7	0.0412 (15)	0.0237 (12)	0.0215 (11)	−0.0049 (11)	0.0100 (10)	−0.0010 (9)
C8	0.0552 (18)	0.0334 (14)	0.0293 (13)	0.0001 (12)	0.0171 (12)	−0.0026 (11)
C9	0.0616 (19)	0.0268 (13)	0.0390 (15)	−0.0123 (13)	0.0160 (14)	−0.0078 (11)
C10	0.0355 (14)	0.0236 (12)	0.0195 (11)	0.0024 (10)	0.0075 (10)	0.0011 (9)
C11	0.0412 (17)	0.0514 (18)	0.0456 (16)	0.0133 (14)	0.0122 (13)	0.0050 (14)
C12	0.0423 (19)	0.066 (2)	0.083 (3)	0.0198 (16)	0.0219 (18)	0.004 (2)
C13	0.0309 (18)	0.073 (3)	0.111 (3)	0.0081 (17)	−0.0148 (19)	−0.020 (2)
C14	0.053 (2)	0.110 (3)	0.071 (2)	0.023 (2)	−0.0308 (19)	−0.039 (2)
C15	0.0443 (18)	0.074 (2)	0.0458 (18)	0.0131 (16)	−0.0064 (14)	−0.0239 (16)

### Geometric parameters (Å, °)

**Table:**

Zn1—O1	2.0540 (19)	C5—C10	1.507 (3)
Zn1—O3	2.0336 (18)	C11—C12	1.377 (4)
Zn1—O4	2.0283 (18)	C12—C13	1.364 (5)
Zn1—N2	2.030 (2)	C13—C14	1.363 (5)
Zn1—O2^i^	2.0450 (19)	C14—C15	1.377 (5)
O1—C7	1.242 (3)	C6—H6A	0.9300
O2—C7	1.238 (4)	C8—H8A	0.9600
O3—C10^ii^	1.246 (3)	C8—H8B	0.9600
O4—C10^iii^	1.240 (3)	C8—H8C	0.9600
O5—N1	1.199 (3)	C8—H8D	0.9600
O6—N1	1.215 (3)	C8—H8E	0.9600
N1—C3	1.490 (3)	C8—H8F	0.9600
N2—C11	1.336 (4)	C9—H9A	0.9600
N2—C15	1.326 (3)	C9—H9B	0.9600
C1—C2	1.400 (3)	C9—H9C	0.9600
C1—C6	1.389 (3)	C9—H9D	0.9600
C1—C7	1.515 (3)	C9—H9E	0.9600
C2—C3	1.393 (3)	C9—H9F	0.9600
C2—C8	1.506 (3)	C11—H11A	0.9300
C3—C4	1.395 (3)	C12—H12A	0.9300
C4—C5	1.406 (3)	C13—H13A	0.9300
C4—C9	1.507 (3)	C14—H14A	0.9300
C5—C6	1.386 (3)	C15—H15A	0.9300
			
O1—Zn1—O3	88.59 (9)	N2—C11—C12	121.7 (3)
O1—Zn1—O4	87.41 (9)	C11—C12—C13	119.1 (3)
O1—Zn1—N2	99.73 (8)	C12—C13—C14	119.3 (3)
O1—Zn1—O2^i^	158.34 (8)	C13—C14—C15	118.9 (3)
O3—Zn1—O4	158.24 (8)	N2—C15—C14	122.2 (3)
O3—Zn1—N2	100.58 (8)	C1—C6—H6A	119.00
O2^i^—Zn1—O3	88.82 (8)	C5—C6—H6A	119.00
O4—Zn1—N2	101.18 (8)	C2—C8—H8A	109.00
O2^i^—Zn1—O4	87.07 (9)	C2—C8—H8B	109.00
O2^i^—Zn1—N2	101.89 (8)	C2—C8—H8C	109.00
Zn1—O1—C7	127.48 (18)	C2—C8—H8D	109.00
Zn1^i^—O2—C7	127.43 (17)	C2—C8—H8E	109.00
Zn1—O3—C10^ii^	129.39 (16)	C2—C8—H8F	109.00
Zn1—O4—C10^iii^	126.61 (17)	H8A—C8—H8B	110.00
O5—N1—O6	124.0 (2)	H8A—C8—H8C	109.00
O5—N1—C3	118.9 (2)	H8B—C8—H8C	109.00
O6—N1—C3	117.0 (2)	H8D—C8—H8E	109.00
Zn1—N2—C11	121.32 (17)	H8D—C8—H8F	110.00
Zn1—N2—C15	120.00 (19)	H8E—C8—H8F	109.00
C11—N2—C15	118.7 (2)	C4—C9—H9A	109.00
C2—C1—C6	120.2 (2)	C4—C9—H9B	109.00
C2—C1—C7	122.5 (2)	C4—C9—H9C	110.00
C6—C1—C7	117.2 (2)	C4—C9—H9D	110.00
C1—C2—C3	115.8 (2)	C4—C9—H9E	109.00
C1—C2—C8	122.0 (2)	C4—C9—H9F	109.00
C3—C2—C8	122.3 (2)	H9A—C9—H9B	109.00
N1—C3—C2	116.51 (19)	H9A—C9—H9C	109.00
N1—C3—C4	117.13 (19)	H9B—C9—H9C	110.00
C2—C3—C4	126.3 (2)	H9D—C9—H9E	109.00
C3—C4—C5	115.4 (2)	H9D—C9—H9F	110.00
C3—C4—C9	121.7 (2)	H9E—C9—H9F	109.00
C5—C4—C9	122.9 (2)	N2—C11—H11A	119.00
C4—C5—C6	120.3 (2)	C12—C11—H11A	119.00
C4—C5—C10	122.8 (2)	C11—C12—H12A	120.00
C6—C5—C10	116.71 (19)	C13—C12—H12A	120.00
C1—C6—C5	121.9 (2)	C12—C13—H13A	120.00
O1—C7—O2	126.0 (2)	C14—C13—H13A	120.00
O1—C7—C1	116.4 (2)	C13—C14—H14A	121.00
O2—C7—C1	117.6 (2)	C15—C14—H14A	121.00
O3^iv^—C10—C5	117.08 (19)	N2—C15—H15A	119.00
O4^v^—C10—C5	117.53 (19)	C14—C15—H15A	119.00
O3^iv^—C10—O4^v^	125.3 (2)		
			
O3—Zn1—O1—C7	−70.9 (2)	C15—N2—C11—C12	−2.4 (5)
O4—Zn1—O1—C7	87.7 (2)	C11—N2—C15—C14	0.8 (5)
N2—Zn1—O1—C7	−171.4 (2)	Zn1—N2—C11—C12	177.4 (3)
O2^i^—Zn1—O1—C7	12.3 (4)	Zn1—N2—C15—C14	−179.1 (3)
O1—Zn1—O3—C10^ii^	82.6 (2)	C2—C1—C6—C5	−4.3 (3)
O4—Zn1—O3—C10^ii^	3.2 (4)	C2—C1—C7—O1	74.4 (3)
N2—Zn1—O3—C10^ii^	−177.8 (2)	C7—C1—C2—C8	7.3 (3)
O2^i^—Zn1—O3—C10^ii^	−75.9 (2)	C7—C1—C6—C5	172.4 (2)
O1—Zn1—O4—C10^iii^	−75.2 (2)	C6—C1—C2—C3	3.3 (3)
O3—Zn1—O4—C10^iii^	4.4 (4)	C6—C1—C7—O2	75.5 (3)
N2—Zn1—O4—C10^iii^	−174.7 (2)	C7—C1—C2—C3	−173.3 (2)
O2^i^—Zn1—O4—C10^iii^	83.8 (2)	C6—C1—C2—C8	−176.2 (2)
O1—Zn1—N2—C11	−106.3 (2)	C6—C1—C7—O1	−102.3 (3)
O1—Zn1—N2—C15	73.6 (2)	C2—C1—C7—O2	−107.9 (3)
O3—Zn1—N2—C11	163.4 (2)	C8—C2—C3—C4	179.4 (2)
O3—Zn1—N2—C15	−16.8 (2)	C8—C2—C3—N1	−2.9 (3)
O4—Zn1—N2—C11	−17.0 (2)	C1—C2—C3—C4	0.0 (3)
O4—Zn1—N2—C15	162.8 (2)	C1—C2—C3—N1	177.59 (19)
O2^i^—Zn1—N2—C11	72.3 (2)	N1—C3—C4—C9	2.4 (3)
O2^i^—Zn1—N2—C15	−107.8 (2)	C2—C3—C4—C5	−2.2 (3)
O1—Zn1—O2^i^—C7^i^	−3.3 (4)	C2—C3—C4—C9	−180.0 (2)
O3—Zn1—O2^i^—C7^i^	79.9 (2)	N1—C3—C4—C5	−179.84 (19)
O4—Zn1—O2^i^—C7^i^	−78.7 (2)	C3—C4—C5—C6	1.3 (3)
N2—Zn1—O2^i^—C7^i^	−179.5 (2)	C9—C4—C5—C6	179.0 (2)
Zn1—O1—C7—O2	−11.5 (4)	C3—C4—C5—C10	176.5 (2)
Zn1—O1—C7—C1	166.09 (17)	C9—C4—C5—C10	−5.8 (3)
Zn1^i^—O2—C7—O1	8.2 (4)	C10—C5—C6—C1	−173.6 (2)
Zn1^i^—O2—C7—C1	−169.35 (16)	C4—C5—C10—O3^iv^	138.0 (2)
Zn1—O3—C10^ii^—C5^ii^	169.14 (16)	C4—C5—C10—O4^v^	−44.5 (3)
Zn1—O3—C10^ii^—O4^i^	−8.1 (4)	C6—C5—C10—O3^iv^	−46.6 (3)
Zn1—O4—C10^iii^—O3^i^	−8.2 (4)	C6—C5—C10—O4^v^	130.9 (2)
Zn1—O4—C10^iii^—C5^iii^	169.07 (16)	C4—C5—C6—C1	1.9 (3)
O6—N1—C3—C2	−108.6 (3)	N2—C11—C12—C13	2.5 (5)
O5—N1—C3—C4	−111.8 (3)	C11—C12—C13—C14	−0.8 (6)
O6—N1—C3—C4	69.3 (3)	C12—C13—C14—C15	−0.8 (6)
O5—N1—C3—C2	70.4 (3)	C13—C14—C15—N2	0.8 (6)

Symmetry codes: (i) −*x*+1, −*y*, −*z*+1; (ii) −*x*+1, *y*−1/2, −*z*+3/2; (iii) *x*, −*y*+1/2, *z*−1/2; (iv) −*x*+1, *y*+1/2, −*z*+3/2; (v) *x*, −*y*+1/2, *z*+1/2.
